# Indications, feasibility, safety, and efficacy of CyberKnife radiotherapy for the treatment of olfactory groove meningiomas: a single institutional retrospective series

**DOI:** 10.1186/s13014-020-01506-6

**Published:** 2020-03-12

**Authors:** Jianmin Liu, Rafael Rojas, Fred C. Lam, Farhan A. Mirza, Anand Mahadevan, Ekkehard M. Kasper

**Affiliations:** 1 Department of Neurosurgery, The First Affiliated Hospital of Guangzhou University of Traditional Chinese Medicine, Guangzhou, China; 2 Department of Radiology, Division of Neuroradiology, Beth Israel Deaconess Medical Center, Harvard Medical School, Boston, MA USA; 3 Division of Neurosurgery, Department of Surgery, Hamilton General Hospital, McMaster University Faculty of Health Sciences, 237 Barton Street East, Hamilton, ON L2L 8X8 Canada; 4 Division of Neurosurgery, Kentucky Neuroscience Institute, University of Kentucky, Lexington, KT USA; 5 Department of Radiation Oncology, Geisinger Medical Group, Danville, PA USA

**Keywords:** Olfactory groove meningiomas, CyberKnife radiotherapy, Stereotactic radiosurgery, Fractionated stereotactic radiotherapy; Hypofractionated stereotactic radiotherapy

## Abstract

**Purpose:**

To assess the safety and efficacy of CyberKnife® radiotherapy (CKRT) for the treatment of olfactory groove meningiomas (OGMs).

**Methods:**

A retrospective review was performed of 13 patients with OGM treated with CKRT from September 2005 to May 2018 at our institution. Nine patients were treated primarily with CKRT, 3 for residual disease following resection, and 1 for disease recurrence.

**Results:**

Five patients were treated with stereotactic radiosurgery (SRS), 6 with hypofractionated stereotactic radiotherapy (HSRT), and 2 with fractionated stereotactic radiotherapy (FSRT). The median tumor volume was 8.12 cm^3^. The median prescribed dose was 14.8 Gy for SRS, 27.3 Gy for HSRT, and 50.2 Gy for FSRT. The median maximal dose delivered was 32.27 Gy. Median post treatment follow-up was 48 months. Twelve of 13 patients yielded a 100% regional control rate with a median tumor volume reduction of 31.7%. Six of the 12 patients had reduced tumor volumes while the other 6 had no changes. The thirteenth patient had significant radiation-induced edema requiring surgical decompression. Twelve patients were alive and neurologically stable at the time of the review. One patient died from pneumonia unrelated to his CKRT treatment.

**Conclusions:**

CKRT appears to be safe and effective for the treatment of OGMs.

## Introduction

Olfactory groove meningiomas (OGMs) originate from arachnoid cap cells of the cribriform plate and frontoethmoidal suture in the anterior cranial fossa [1, 2] and contribute to approximately 4–13% of intracranial meningiomas [3]. Treatment options for OGMs include: Observation; open microsurgery [4] or transnasal endoscopic surgery; and radiotherapy including Gamma Knife [5] and linear accelerator (LINAC) [6]. CKRT can be delivered as stereotactic radiosurgery (SRS), fractionated stereotactic radiotherapy (FSRT) [7], or hypofractionated stereotactic radiotherapy (HSRT) [8] – the appropriate regimen is chosen depending on the patient’s tumor characteristics and its proximity to surrounding critical structures or vasculature.

OGMs which are symptomatic, larger than 3 cm in diameter, or demonstrate progressive growth on sequential imaging, are considered suitable for surgical treatment [9]. However, treatment algorithms have not been well established for OGMs smaller than 3 cm in diameter that demonstrate continued growth in patients who are high surgical risk, particularly older patients with significant comorbidities which may preclude conventional open surgical intervention. Surgical resection of OGMs can also be complicated by their close proximity to critical neurovascular structures [10]. Despite improvements in microsurgical techniques, post-operative complications including anosmia and visual disturbances have been reported at rates as high as 89.7 and 7.7%, respectively [10]. Since RT has recently emerged as an effective alternate modality for treatment of primary intracranial diseases particularly in the non-surgical and elderly populations or as adjuvant therapy for residual or recurrent disease the question arises whether such therapy is a suitable option for patients with OGMs which require treatment.

Among the RT technologies available, CKRT is a more recent image-guided frameless robotic system, which is designed for SRS and stereotactic radiation therapy (SRT) [11, 12]. The associated 6D skull tracking system enables highly conformal intracranial high-dose tumor targeting, with sub-millimeter precision in beam delivery allowing for steep dose gradients achievable around the contoured tumor. An additional benefit is the frameless nature of the system which makes it easy to opt for hypofractionation regimens to minimize toxicity, particularly when radiation tolerance of adjacent organs-at-risk is paramount [7]. Therefore, CKRT can be considered an attractive and reasonable primary treatment strategy for OGMs that have been diagnosed in patients who are not suitable candidates for open surgery or for patients in the adjuvant setting, when treatment is needed for postoperative residual or recurrent disease. In the present study, we report our institutional experience over a 10-year period assessing the safety and efficacy of CKRT for the treatment of primary, residual, and recurrent OGMs.

## Materials and methods

### Patient characteristics and study design

Institutional IRB approval was obtained prior to commencement of this study. A retrospective chart review and review of all electronic radiographic data was performed of patients with OGM who were treated with CKRT (Accuray, Inc., Sunnyvale, CA) at Beth Israel Deaconess Medical Center (Boston, USA) between Sept 2005 and May 2018. Patients were included if they had an initial diagnosis of OGM based on MRI scanning (T1WI with contrast or contrast-enhanced fat saturation suppression 3D sequences) or if patients had prior confirmed histological diagnosis of OGM from the time of first surgical resection if they subsequently presented with residual or recurrent disease. Small asymptomatic tumors discovered incidentally on neuroimaging were not included in this study but followed conservatively.

Surgical resection was recommended for patients with rapidly progressive vision loss, marked peritumoral edema, or large tumors (diameter > 3 cm or volume > 10 cm^3^), however, older patients (aged ≥65) with significant comorbidities who were deemed high risk surgical candidates were offered CKRT as an alternative.

Patient characteristics are listed in Table 1. Nine females and 4 males (mean age 71.2 years; range, 47–88) were treated with CKRT during this time period with a mean Karnofsky performance score of 89 (range, 50–100). Six patients presented with cranial nerve deficits including anosmia (*N* = 6) and visual deterioration (*N* = 2), while 2 presented with seizures due to cerebral edema and mass effect, and 2 presented with headaches and dizziness. Nine patients were treated with primary CKRT at the time of initial tumor diagnosis; 3 patients were treated with adjuvant CKRT for residual disease following subtotal resection that showed interval growth on at least 2 serial contrast-enhanced MRI scans over a 6-month period; 1 patient was treated for disease recurrence after a resection 10 years prior.

**Table 1:** Clinical characteristics of patients

Characteristic	Total
**Number of patients**	13
**Median age (yrs), (range)**	71.2, (47–88)
**Gender**	
Male	4 (30.8%)
Female	9 (69.2%)
**Mean Karnovsky performance score, (range)**	*89 (50–100)*
**Primary vs. Residual/ Recurrent disease**	
Primary	9
Residual	3
Recurrent	1
**Median tumor volume (cm**^**3**^**), (range)**	10.26 (0.85–43.76)
**Presenting symptoms**	
Asymptomatic	3
Seizure	2
Anosmia	4
Visual deterioration	2
Headaches,dizziness	2

### CKRT dose and fractionation treatment planning

Treatment plans were generated using an inverse planning method by the Accuray MultiPlan treatment software and are summarized in Tables 2 and 3. Prescribed dosing and fractionation schedules were decided by an interdisciplinary team of neurosurgeons and radiation oncologists taking into account the size and volume of the tumor, its proximity to critical neurovascular structures, and previous treatment history. We used Dmax synonymously with the smallest reasonable assessable voxel of 0.03 cc. We used 10 Gy for SRS, 25 Gy in 5 fractions for HSRT and 54 Gy in 30 fractions for FSRT to optic nerves and chiasm. Single fraction SRS was recommended for tumors less than 10 cm^3^ which were not topographically associated with the optic apparatus. Lesions greater than 10 cm^3^ or with very close proximity to critical structures (i.e. optic nerves, optic chiasm, and brainstem) were treated with SRT using either HRST or FSRT. Five out of 13 patients (38.4%) were treated with single fraction SRS, 6 patients (46.2%) received HSRT in 3 to 5 fractions given on consecutive days, and 2 patients (15.4%) received FSRT in 25 to 28 daily fractions over 5 to 6 weeks.

**Table 2:** Summary of patient characteristics and CyberKnife radiotherapy parameters

Patient No.	Gender	Age (yrs)	CKRT regimen	Tumor volume (cm^3^)	Number of fractions	Prescription dose (Gy)	Dose per fraction (Gy)	Maximum dose (Gy)	Prescription isodose (%)	Conformality index	Heterogeneity index	Lesion coverage	Total No. of beams
1	F	47	SRS	3.14	1	15	15	18.52	81%	1.1	1.23	95.30%	190
2	F	72	SRS	6.97	1	15	15	18.29	82%	1.29	1.22	94.60%	230
3	M	57	SRS	8.76	1	13	13	17.33	75%	1.38	1.33	98.80%	262
4	F	75	SRS	0.52	1	13	13	18.57	70%	1.38	1.43	96.95%	150
5	M	71	SRS	3.46	1	18	18	24	75%	1.17	1.33	95.84%	125
6	F	86	HSRT	11.91	3	24	8	30	80%	1.51	1.25	97.43%	268
7	F	79	HSRT	3.73	5	30	6	35.71	84%	1.28	1.19	96.34%	190
8	F	84	HSRT	42.39	5	30	6	36.59	82%	1.22	1.22	95.16%	250
9	F	84	HSRT	10.12	5	30	6	37.97	79%	1.53	1.27	95.01%	209
10	F	63	HSRT	0.44	5	25	5	32.89	76%	1.77	1.32	93.48%	91
11	M	88	HSRT	11.35	5	25	5	33.33	75%	1.26	1.33	92.69%	157
12	M	60	FSRT	2.41	25	50	2	55.56	90%	1.95	1.11	95.35%	143
13	F	60	FSRT	0.32	28	50.4	1.8	60.72	83%	1.65	1.2	96.65%	180

**Table 3:** Characteristics of CKRT treatment plans

Characteristics	SRS (*n* = 5)	HSRT (*n* = 6)	FSRT (*n* = 2)	All patients (*n* = 13)
**Tumor Volume (cm**^**3**^**)**	4.57 (0.52–8.76)	13.33 (0.44–42.39)	1.37 (0.32–2.41)	8.12 (0.32–42.39 cc)
**Number of Fractions**	1	3–5	25–28	1–28
**Prescription Dose (Gy)**	14.8 (13–18)	27.3 (24–30)	50.2 (50–50.4)	13–50.4
**Dose Fraction (Gy)**	14.8 (13–18)	6 (5–8)	1.9 (1.8–2)	1.8–18
**Prescription Isodose (%)**	76.7% (70–82%)	79.3% (75–84%)	86.5% (83–90%)	79.38% (70–90%)
**Maximum Dose (Gy)**	19.34 (17.33–24)	34.42 (30–37.97)	58.14 (55.56–60.72)	32.27 (17.33–60.72)
**Lesion Coverage**	96.3% (95.3–98.8%)	95.03% (92.69–97.43%)	96% (95.35–96.65%)	95.66% (92.69–98.8%)
**Conformality index**	1.26 (1.1–1.38)	1.43 (1.22–1.77)	1.8 (1.69–1.95)	1.43 (1.1–1.95)
**Total Beam**	191 (125–262)	194 (91–268)	161 (143–180)	91–268

**Fig. 1:**
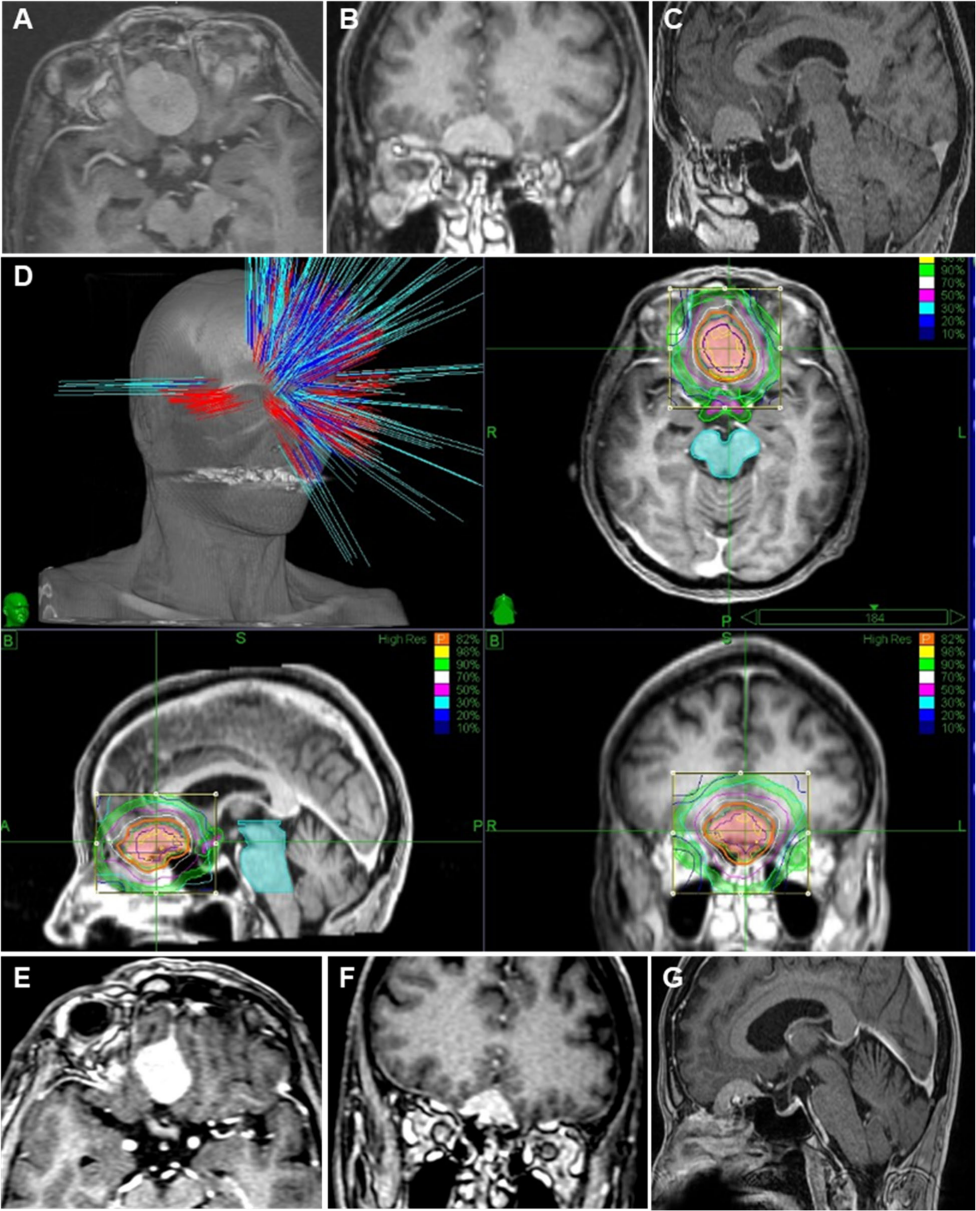
CyberKnife radiotherapy for the treatment of olfactory groove meningiomas. Representative **a**) axial, **b**) coronal, and **c**) sagittal MRI images of a patient with an olfactory groove meningioma with a maximal diameter of 3.5 cm. **d** CyberKnife surgical planning schematic with isodose lines around the patient’s tumor. **e**, **f**, **g** MRI images of the same patient showing decreased tumor size (maximal diameter 2.9 cm) 6 years following CKRT

The median tumor volume was 4.57 cm^3^ (range, 0.85–8.76 cm^3^) for SRS, 13.33 cm^3^ (range, 0.44–42.39 cm^3^) for HSRT, and 1.37 cm^3^ (range, 0.32–2.41) for FSRT. HSRT was given in 3 or 5 fractions of 8 Gy and 5 Gy per fraction respectively, and FSRT was given in 25–28 fractions at 1.8 or 2 Gy per fraction. The median prescription dose was 14.8 Gy (range, 13–18 Gy) delivered to the 70–82% isodose line for SRS with an average of 191 beams (range, 99–262 beams); 27.3 Gy (range, 24–30 Gy to the 75–84% isodose line for HSRT with an average of 194 beams (range, 91–268 beams); and 50.2 Gy (range, 40–50.4 Gy) to the 83–90% isodose line for FSRT with an average of 161 beams (range, 143–180). The median tumor target volume coverage to the full prescription dose was 95.66% (range, 92.69–98.8%), which was achieved with a median conformality index of 1.43 (range, 1.1–1.95) for a tumor volume of 8.12 cm^3^ (range, 0.32–42.39 cm^3^). The median maximum delivered dose was 32.27 Gy (range, 17.33–60.72 Gy). The proximity and abutment to the optic pathway was more critical in the choice of SRT, more importantly than volume. Tumors abutting the optic pathway were more likely to receive FSRT even when smaller, whereas larger tumors close but away from the optic pathway tended to receive HSRT when large (as opposed to SRS for smaller lesions). Representative images of a patient with an OGM and the treatment planning used to treat the lesion are shown in Fig. 1.

### Radiosurgery preparation

All patients were treated using the CyberKnife® Robotic Radiosurgery System (Accuray, Inc., Sunnyvale, CA) with frameless skull base real time image-guided tracking. Pretreatment 1.25 mm thin-slice, high-resolution computer tomographic scans and matching gadolinium-enhanced magnetic resonance imaging (MRI) scans (MPRAGE sequence) were acquired and fused for treatment planning. Treatment plans were generated using an inverse planning method by the Accuray MultiPlan treatment software. An example of a treatment plan from patient #2 is shown in Fig. 1. Doses were prescribed to the isodose surface that encompassed the margin of the tumor. Patients received 4 mg of dexamethasone immediately after each treatment. For multisession treatments, the typical time interval between fractions was 24 h. The quality of treatment plans was assessed by evaluating target coverage, dose heterogeneity, and conformity. Digitally reconstructed radiograms were computationally synthesized to allow near real-time patient tracking throughout radiosurgery. Treatment safety and efficacy were determined by: 1) Quantifying changes in tumor volume on subsequent MRI scans at the end of each patient’s follow-up period, and 2) Assessment of preservation of optic and olfactory nerve functions.

### Follow-up, toxicity, and statistical analysis

Patients were seen at 1, 3, 6, and 12 months after radiotherapy and then annually for the next 5 years and bi-annually thereafter. A contrast-enhanced MRI brain scan was obtained, and a complete neurological exam with particular focus on visual and olfactory function was performed at each visit. Local control was defined by tumors with less than 2 mm change in size in any dimension measured in the longest diameter on two sequential MRI scans.

Patients were monitored for radiation-induced adverse events both clinically and radiologically. Acute toxicity was defined as changes in signs or symptoms occurring within 3 months of radiotherapy that were considered likely to be related to radiotherapy. Long-term toxicity was defined as any such events occurring or persisting beyond 3 months following radiotherapy. Toxicities were evaluated according to the Common Terminology Criteria for Adverse Events, version 4.0 [13].

Local control rates were calculated from the date of the first SRS treatment to the last MRI scan that showed tumor changes. Overall survival was calculated from the date of the initial SRS treatment to the last date of clinical follow-up.

## Results

### Local tumor control

One patient died from pneumonia 1 month after CKRT for reasons unrelated to radiotherapy and the patient was thus excluded from our outcome analysis. Of the remaining 12 patients, the median following-up was 48 months. Details of local control are shown in Table 4. Radiographic local tumor control was maintained in 100% of patients at the time of their last follow-up MRI scan. Tumors decreased in size after treatment in 6 (50%) patients and remained stable in the remaining 6 (50%) patients. Figure 1 shows pre- (Fig. 1a, b, and c) and post-treatment (Fig. 1e, f, and g) MRI scans in a representative patient (patient #2) with an OGM along with treatment planning and isodose lines for CKRT (Fig. 1d). The median tumor size in our cohort decreased from 2.52 cm to 2.22 cm with a relative linear size reduction of 11.9% corresponding to a median volume decrease of 31.7%.

**Table 4:** Clinical outcomes after CKRT

Patient No.	Tumor type	RT type	Follow up (mos.)	CKRT symptoms	Pre−/Post-CKRT peritumoral edema	Visual function post-CKRT	Olfaction function post-CKRT	Change in tumor size Post-CKRT	Pre−/Post CKRT KPS	Pre−/Post- CKRT median tumor size (cm)
1	Primary	SRS	9	None	Mild/Slightly increased	Unchanged	Unchanged	Unchanged	50/50	2.50/2.50
2	Primary	SRS	106	Fatigue	None/None	Unchanged	Unchanged	Decreased	90/100	3.50/2.90
3	Primary	SRS	10	None	Moderate/Significantly increased	Unchanged	Unchanged	Unchanged	90/80	2.80/2.9
4	Primary	SRS	45	None	None/None	Unchanged	Unchanged	Unchanged	100/90	1.15/1.06
5	Residual	SRS	60	None	None/None	Unchanged	Unchanged	Unchanged	90/90	1.80/1.8
6	Primary	HSRT	99	Fatigue, forgetfulness, hallucinations	Mild / Minimally increased	Unchanged	Unchanged	Unchanged	90/70	2.56/2.49
7	Primary	HSRT	11	None	Moderate/Minimally increased	Unchanged	Unchanged	Unchanged	80/80	4.49/4.41
8	Primary	HSRT	89	Fatigue	Moderate/Minimally increased	Unchanged	Unchanged	Decreased	80/70	3.00/2.60
9	Primary	HSRT	47	None	None/None	Unchanged	Unchanged	Decreased	100/90	1.30/0.93
10	Residual	HSRT	20	None	Mild/Mild	Improved	Unchanged	Decreased	90/70	3.91/3.33
11	Residual	FSRT	19	Headaches, fatigue, forgetfulness	None/None	Unchanged	Unchanged	Decreased	100/90	2.00/1.70
12	Recurrent	FSRT	61	None	without /without	Unchanged	unchanged	Decreased	100/90	1.20/0

### Radiation-associated toxicities

Radiation-associated toxicities are shown in Table 4. Patients received CKRT treatments ranging from a single to 28 consecutive fractions (Table 3). Patients were assessed before, during, and after radiation therapy for changes in vision, olfaction, and other neurological symptoms. Of the 6 patients who had presented with pre-existing olfactory deficits, symptoms remained unchanged throughout their course of treatment. Of the 4 patients with visual deficits, 3 remained unchanged while one patient showed improved visual fields.

Four patients (1 SRS, 2 HSRT, and 1 FSRT) had CTAE v.4.0 Grade 1 radiation-induced adverse effects including headaches, fatigue, forgetfulness, and hallucinations, all of which were lasting less than 6 weeks and did not require specific interventions. Patients without pre-treatment peritumoral edema (*N* = 6) did not demonstrate any post-treatment radiation-associated peritumoral edema. Six patients had pre-existing tumor edema, of whom four (1 SRS, 3 HRST) demonstrated transiently increased treatment-associated edema lasting no more than 9 weeks following CKRT, all of which responded well to a short course of steroids (Dexamethasone 2 mg P.O. TID × 2 weeks). The edema was not associated with changes in tumor volume or hemorrhage. One SRS patient, with a tumor volume of 8.76 cm^3^ and moderate pre-treatment peritumoral edema (Table 2, patient #3), demonstrated significant post-treatment peritumoral edema with associated local mass effect following CKRT which did not respond well to steroid treatment, ultimately requiring open surgical resection of his tumor, with complete resolution of the edema 2 weeks following the surgery.

### Survival

Apart from the 1 patient (Table 2, patient #6) who passed away from pneumonia not associated with the CKRT treatment, all patients (100%) were alive at their last follow-up. Given our relatively short median follow-up of 48 months, we are continuing to follow this cohort in hopes of obtaining longer-term survival data.

## Discussion

### Indications for using CyberKnife radiation therapy for the treatment of olfactory groove meningiomas

To the best of our knowledge, this is the first article to specifically review the indications, safety, and efficacy of using CyberKnife for the treatment of OGMs. There have been a mere handful of studies assessing the use of radiotherapy for the treatment of OGMs due to the relatively low incidence of disease and the relatively recent advent of radiosurgery. Two recent retrospective studies reported the safety and efficacy of Gamma Knife (GK) [5] and Novalis LINAC [6] radiosurgery using SRS or FSRT for OGMs (Table 5). GK can cause patient discomfort due to the invasive head frame required to immobilize the skull and prolonged fractionated regimens makes frame fixation uncomfortable and impractical [14], however a frameless system has recently been introduced for clinical use, which may allow for improved patient comfort and geometric dose accuracy. LINAC systems allow for SRT using a non-invasive head fixation, however, the frame does not provide near-perfect rigid fixation and therefore target volumes require a small added margin around the tumor volume to account for day-to-day imprecision in geometric target positioning. This adjustment is needed to minimize the chance of missing part of the tumor. Furthermore, radiation-induced adverse events such as anosmia and visual deterioration have been reported to be as high as 7.3–14.3% using GK or LINAC [5, 6].

**Table 5:** Summary of radiotherapy studies for the treatment of olfactory groove meningioms

Study	Radiotherapy Technique (No. of Patients)	Median Tumor Volume (cm^3^), (range)	Number of Fractions	Median Prescribed Dose (Gy), (range)	Dose Per Fraction (Gy)
Zaorsky et al. 2014 [6]	SRS (7)	2.90 (1.30–6.20)	1	16.1 (15–18)	15–18
	FSRT (7)	3.03 (1.13–7.10)	25–30	52.9 (50.0–56.0)	1.8–2.0
	(GK or LINAC)				
Gande et al. 2014 [5]	SRS (41)	8.5 (0.6–56.1)	1	13 (10–20).	10–20
	(GK)				
Present Study	SRS (5)	4.57 (0.52–8.76)	1	14.8 (13.0–18.0)	13–18
	HSRT (6)	13.33 (0.44–42.39)	3–5	27.3 (24–30)	5–8
	FSRT (2)	1.37 (0.32–2.41)	25–28	50.2 (50–50.4)	1.8–2
	(CKRT)				

The CyberKnife system allows for the delivery of 6 MV photons for radiotherapy via an image-guided radiosurgical treatment platform with a frameless robotic manipulator and 6D Skull Tracking system to highly conformal intracranial high-dose tumor targeting, with sub-millimeter precision beam delivery and steep dose gradients achievable around the contoured target volume [11, 12, 15]. These advantages have allowed CKRT to be a feasible option for patients who are not considered suitable for surgery or cannot tolerate frame-based RT. Based on our institutional experience and our review of pertinent literature on the treatment of skull base meningiomas, we propose four main indications for which CKRT may be considered as a primary or secondary treatment strategy for OGMs. These include: 1) Patients who are not suitable surgical candidates, especially older patients with significant comorbidities (i.e. cardiovascular disease or prior stroke on anticoagulation, patients with brittle diabetes or those with significant pulmonary or end-stage renal disease) [16]; 2) Tumors less than 10 cm^3^ in size with progressive symptoms or continuous growth in elderly patients [17]; 3) Subtotally resected residual or recurrent OGMs demonstrating growth in patients who are not surgical candidates or do not wish to pursue second surgical intervention [15, 17, 18]; and 4) patients with residual OGMs diagnosed with high WHO grade pathology [12, 15, 18].

SRS can be considered suitable for tumors that are limited in size up to 3 cm in maximal diameter or 10 cm^3^ in volume with distinct margins, limited mass effect, minimal to no surrounding edema, with a sufficient distance of 3 to 5 mm from nearby critical organs at risk to allow for appropriate tissue sparing via dose restriction [15, 17, 19, 20]. In our case series, 5 of 13 patients with OGMs whose median tumor volume was 4.57 cm^3^ safely received single fraction SRS with a median prescribed dose of 14.8 Gy.

SRT (HRST and FSRT) delivered at lower doses over several fractions can be used for tumors that are within close proximity to critical neurovasculature structures to achieve tissue sparing of radiation-associated adverse events [7, 8]. In addition, SRT can be used to treat large OGMs (> 10 cm^3^) that may be in close proximity to the optic apparatus [8, 11, 18, 21]. In our case series, 4 patients with smaller sized OGMs within close proximity to the optic chiasm and 4 patients with larger OGMs with a median tumor volume of 10.1 cm^3^ were treated with SRT. In general, HSRT delivered over 2 to 5 fractions is significantly more convenient than the 25–28 fractions delivered over 4 to 6 weeks required for FRST, however FSRT has been found to have a radiobiological advantage in sparing late-responding tissues [7, 11, 18]. The median treated tumor volume (8.12 cm^3^) in our cohort was similar compared to that in the series reported by Gande et al. using GK (median 8.5 cm^3^) [5] but was significantly larger compared to the cohort reported by Zaorsky and colleagues using LINAC (2.97 cm^3^) [6]. Our results suggest that CKRT is also well tolerated and can be used to treat patients with larger OGMs.

### Tumor control using CyberKnife radiotherapy

Pre-treatment tumor volume is the most important factor determining tumor control rates in patients treated with RT [5]. As mentioned above, the mean tumor volume before CKRT was larger in our case series compared to the cohort treated by Zaorsky et al. using GK and LINAC RT [6]. With the caveats of a relatively short mean follow-up of 48 months and the exclusion of the one patient who passed away from non-RT-associated pneumonia, we observed a 100% local control rate over the observation period with a decrease in median tumor size from 2.52 cm to 2.22 cm corresponding to a relative size reduction of 11.9% in the longest linear axis of the tumor and 31.7% reduction in tumor volume. Of note, we found that the 4 patients with the largest tumors (with volumes > 10 cm^3^) treated with SRT were equally well controlled than patients with smaller tumors. Local control rates were therefore consistent with previous reports using GK or LINAC [5, 6]. There appeared to be a trend towards a higher rate of radiographic reduction in tumor volume in our patients using CKRT (50%) which was relatively higher than that previously reported using SRS (see Table 6), although a larger number of patients would need to be enrolled and treated in the future in order to statistically confirm the higher efficacy of CKRT in this cohort. Zaorsky et al. reported 14 cases of OGMs treated with GK or LINAC, of which only 3 cases (21%) displayed tumor shrinkage [6]. Similarly, Gande et al. in their series of 41 cases demonstrated that only thirteen patients (32%) showed post-SRS tumor regression with 2 patients showing further tumor progression despite treatment [5]. Our results suggest that CKRT delivered as SRS or SRT may achieve equal or better local tumor control than GK or LINAC RT. Increased sample size with longer term follow-up will help to investigate our claims.

**Table 6:** Comparison of safety and efficacy of radiotherapy studies for the treatment of olfactory groove meningiomas

Study	No. of Patients	Median Age (yrs), (range)	RT Technique	Deterioration in Olfaction post-RT (%)	Deterioration in Vision post-RT (%)	Tumor Control (%)	Patients with Tumor Reduction (%)	Median Follow-up (mos.),(range)
Zaorsky et al. 2014 [6]	14	57 (50–73)	SRS & FSRT using GK or LINAC	0	14.3	100	21	64 (21–125)
Gande et al. 2014 [5]	41	58 (40–87)	SRS Using GK	0	7.3	95	32	76 (7–194)
Present Study	12	71.2 (47–88)	SRS, HSRT, & FSRT Using CKRT	0	0	100	50	48 (9–106)

### Radiation toxicities associated with CyberKnife radiotherapy

The frequency of RT-induced post-treatment edema has been reported to be between 6 and 43% for intracranial meningioma radiosurgery [15, 22]. Zaorsky et al. reported two cases of treatment-associated acute peritumoral edema in their study of 14 patients [6]. In our current study, we observed minor treatment-associated peritumoral edema in 5 of 12 patients (Table 4). Factors that make a patient more prone to developing post-SRT treatment edema for the treatment of skull-based meningiomas include: Tumor volumes > 10 cm^3^; pre-existing peritumoral edema prior to receiving RT; and age > 60 years [15, 17]. We attributed our increased incidence of post-CKRT edema to the fact that our patients already had a high incidence of pre-treatment peritumoral edema (6/13), relatively larger tumors volumes at the time of treatment compared to that reported by Zaorsky et al. (median volume 8.12 vs 3.57 cm^3^), and that our patients were significantly older (median age 71.2 years vs 57 years). Our one patient with an 8.76 cm^3^ tumor and moderate peritumoral edema, who experienced significant SRS-associated edema requiring surgical decompression highlights the need for careful selection and treatment planning to minimize these adverse events and ensure patient safety.

Despite the incidence of CKRT-associated edema, 100% of our patients had stable visual and olfactory function at the time of last follow-up, compared to 7.3 and 14.3% visual impairment following GK or LINAC reported by Gande et al. [5] and Zaorsky et al. [6], respectively, and highlights the advantages of using fractionated regimens for cranial nerve sparing.

### Limitations of the study

This is a single center retrospective cohort study for which the number of patients is a relatively small and the median length of follow-up is relatively short. However, given the increasing availability of frameless technology provided by CKRT, we hope that our study provides initial data to support CKRT as an available alternate treatment to surgery which is needed in a select patient population not suitable for microsurgery. Our experience with CKRT for OGMs is consistent with other published studies using GK and LINAC RT. These small cohort studies warrant more extensive studies in multicenter trials to reach cohort numbers large enough to establish a preferred dosing regimen in this setting.

## Conclusion

In summary, this study suggests that CKRT is a safe and effective way of treating de novo, residual, and recurrent OGMs with high rates of local control. Single fraction SRS can be used to treat small to moderate sized OGMs (< 10 cm^3^) that are not spatially associated with the optic apparatus while larger OGMs (> 10 cm^3^) can be treated using fractionated planning to achieve normal tissue sparing and preserve cranial nerve function. Longer term follow-up with increased patient accrual will increase the significance of our findings.

## Data Availability

We will make data and materials available if needed.

## Abbreviations

CKRT: CyberKnife radiation therapy
FSRT: Fractionated stereotactic radiotherapy
GK: Gamma Knife
HSRT: Hypofractionated stereotactic radiotherapy
LINAC: Linear accelerator
OGM: Olfactory groove meningioma
RT: Radiotherapy
SRS: Stereotactic radiosurgery
SRT: Stereotactic radiotherapy

## References

[1] Mirimanoff RO, Dosoretz DE, Linggood RM, Ojemann RG, Martuza RL (1985). Meningioma: analysis of recurrence and progression following neurosurgical resection. J Neurosurg.

[2] Kepes JJ (1982). Meningiomas: biology, pathology, and differential diagnosis (Masson monographs in diagnostic pathology).

[3] Adappa ND, Lee JY, Chiu AG, Palmer JN (2011). Olfactory groove meningioma. Otolaryngol Clin N Am.

[4] Bitter AD, Stavrinou LC, Ntoulias G, Petridis AK, Dukagjin M, Scholz M (2013). The role of the Pterional approach in the surgical treatment of olfactory groove Meningiomas: a 20-year experience. J Neurol Surg B Skull Base.

[5] Gande A, Kano H, Bowden G, Mousavi SH, Niranjan A, Flickinger JC (2014). Gamma knife radiosurgery of olfactory groove meningiomas provides a method to preserve subjective olfactory function. J Neuro-Oncol.

[6] Zaorsky NG, Andrews DW, Podrat J, Gunn V, Liu H, Werner-Wasik M (2014). Radiotherapy as primary treatment of olfactory groove meningiomas is associated with preserved cranial nerve function and excellent quality of life. Austin J Surg.

[7] Conti A, Pontoriero A, Midili F, Iati G, Siragusa C, Tomasello C (2015). CyberKnife multisession stereotactic radiosurgery and hypofractionated stereotactic radiotherapy for perioptic meningiomas: intermediate-term results and radiobiological considerations. Springerplus.

[8] Demiral S, Dincoglan F, Sager O, Gamsiz H, Uysal B, Gundem E (2016). Hypofractionated stereotactic radiotherapy (HFSRT) for who grade I anterior clinoid meningiomas (ACM). Jpn J Radiol.

[9] Pallini R, Fernandez E, Lauretti L, Doglietto F, D'Alessandris QG, Montano N (2015). Olfactory groove meningioma: report of 99 cases surgically treated at the Catholic University School of Medicine, Rome. World Neurosurgery.

[10] Fountas KN, Hadjigeorgiou GF, Kapsalaki EZ, Paschalis T, Rizea R, Ciurea AV (2018). Surgical and functional outcome of olfactory groove meningiomas: lessons from the past experience and strategy development. Clin Neurol Neurosurg.

[11] Di Franco R, Borzillo V, Ravo V, Falivene S, Romano FJ, Muto M (2018). Radiosurgery and stereotactic radiotherapy with cyberknife system for meningioma treatment. Neuroradiol J.

[12] Zhang M, Ho AL, D'Astous M, Pendharkar AV, Choi CY, Thompson PA (2016). CyberKnife stereotactic radiosurgery for atypical and malignant Meningiomas. World Neurosurgery.

[13] Institute NC. Common terminology criteria for adverse events. Version 4.0. Available from: http://evs.nci.nih.gov/ftp1/CTCAE/CTCAE_4.03_2010-06-14_QuickReference_5x7.pdf. Accessed 25 Dec 2019.

[14] Andrews DW, Bednarz G, Evans JJ, Downes B (2006). A review of 3 current radiosurgery systems. Surg Neurol.

[15] Morimoto M, Yoshioka Y, Shiomi H, Isohashi F, Konishi K, Kotsuma T (2011). Significance of tumor volume related to peritumoral edema in intracranial meningioma treated with extreme hypofractionated stereotactic radiation therapy in three to five fractions. Jpn J Clin Oncol.

[16] Kaul D, Budach V, Graaf L, Gollrad J, Badakhshi H (2015). Outcome of elderly patients with meningioma after image-guided stereotactic radiotherapy: a study of 100 cases. Biomed Res Int.

[17] Vera E, Iorgulescu JB, Raper DM, Madhavan K, Lally BE, Morcos J (2014). A review of stereotactic radiosurgery practice in the management of skull base meningiomas. J Neurol Surg B Skull Base.

[18] Minniti G, Amichetti M, Enrici RM (2009). Radiotherapy and radiosurgery for benign skull base meningiomas. Radiat Oncol.

[19] Girkin CA, Comey CH, Lunsford LD, Goodman ML, Kline LB (1997). Radiation optic neuropathy after stereotactic radiosurgery. Ophthalmology.

[20] Starke RM, Przybylowski CJ, Sugoto M, Fezeu F, Awad AJ, Ding D (2015). Gamma knife radiosurgery of large skull base meningiomas. J Neurosurg.

[21] Kondziolka D, Flickinger JC, Lunsford LD (2008). The principles of skull base radiosurgery. Neurosurg Focus.

[22] Novotny J, Kollova A, Liscak R (2006). Prediction of intracranial edema after radiosurgery of meningiomas. J Neurosurg.

